# Strigolactones enhance root‐knot nematode (*Meloidogyne graminicola*) infection in rice by antagonizing the jasmonate pathway

**DOI:** 10.1111/nph.15953

**Published:** 2019-07-02

**Authors:** Zobaida Lahari, Chhana Ullah, Tina Kyndt, Jonathan Gershenzon, Godelieve Gheysen

**Affiliations:** ^1^ Department of Biotechnology Ghent University Ghent 9000 Belgium; ^2^ Department of Biochemistry Max Planck Institute for Chemical Ecology Jena 07745 Germany

**Keywords:** jasmonic acid, *Oryza sativa*, phytohormone signaling, plant–nematode interaction, strigolactone

## Abstract

- Strigolactones (SLs) are carotenoid‐derived plant hormones that also act in the rhizosphere to stimulate germination of root‐parasitic plants and enhance plant symbiosis with beneficial microbes. Here, the role of SLs was investigated in the interaction of rice (*Oryza sativa*) roots with the root‐knot nematode *Meloidogyne graminicola*.
- Genetic approaches and chemical sprays were used to manipulate SL signaling in rice before infection with *M. graminicola*. Then, nematode performance was evaluated and plant defense hormones were quantified.
- *Meloidogyne graminicola* infection induced SL biosynthesis and signaling and suppressed jasmonic acid (JA)‐based defense in rice roots, suggesting a potential role of SLs during nematode infection. Whereas the application of a low dose of the SL analogue GR24 increased nematode infection and decreased jasmonate accumulation, the SL biosynthesis and signaling *d* mutants were less susceptible to *M. graminicola*, and constitutively accumulated JA and JA‐isoleucine compared with wild‐type plants. Spraying with 0.1 μM GR24 restored nematode susceptibility in SL‐biosynthesis mutants but not in the signaling mutant. Furthermore, foliar application of the SL biosynthesis inhibitor TIS108 impeded nematode infection and increased jasmonate levels in rice roots.
- In conclusion, SL signaling in rice suppresses jasmonate accumulation and promotes root‐knot nematode infection.

## Introduction

Strigolactones (SLs) are carotenoid‐derived plant hormones originally identified as signaling molecules in the rhizosphere (Cook *et al*., 1966; Akiyama *et al*., 2005; Umehara *et al*., 2008). The first known SL, strigol, was isolated from cotton root exudates, and detected as a stimulant of seed germination of the root‐parasitic weed *Striga lutea* (Cook *et al*., 1966). To date, > 20 naturally occurring SLs have been identified in different plant species, which have been shown to stimulate seed germination of various root‐parasitic plants (Al‐Babili & Bouwmeester, 2015). The precursor of SL biosynthesis is carlactone, which is derived from *trans*‐β‐carotene via the action of the isomerase DWARF27 (D27) and then a sequential oxidative cleavage by the carotenoid cleavage dioxygenase enzymes (CCD7 and CCD8) in plastids (Alder *et al*., 2012). Carlactone is then transported into the cytoplasm where a MAX1‐type monooxygenase transforms it into carlactonic acid that is eventually converted into SLs and SL‐like compounds (Seto *et al*., 2014). In rice (*Oryza sativa*), the genes *OsD17* and *OsD10* encode the enzymes CCD7 and CCD8, respectively. The genes encoding the α/β‐fold hydrolase D14 and the F‐box protein D3 are known as SL‐signaling genes in rice (Cheng *et al*., 2013). The SL rice mutants are commonly known as *dwarf* (*d*) or *high‐tillering dwarf* (*htd*) mutants (Gomez‐Roldan *et al*., 2008; Umehara *et al*., 2008).

SLs play a pivotal role in various aspects of plant growth and development as well as in plant symbiosis with beneficial microbes (Akiyama *et al*., 2005; Gomez‐Roldan *et al*., 2008; Kapulnik *et al*., 2011). As endogenous signaling molecules, SLs have been shown to modulate plant architecture. For example, SL biosynthesis or signaling mutants of different plant species showed increased numbers of lateral shoot branches (Gomez‐Roldan *et al*., 2008; Umehara *et al*., 2008). SLs also promote primary root growth and inhibit the formation of lateral and adventitious roots in *Arabidopsis* (Kapulnik *et al*., 2011; Ruyter‐Spira *et al*., 2011). In rice, SL‐biosynthesis and ‐signaling mutants exhibit shorter crown roots (Arite *et al*., 2012). Furthermore, SLs negatively regulate mesocotyl elongation in darkness (Hu *et al*., 2013).

As rhizosphere signaling molecules, SLs promote plant association with mycorrhizas by stimulating hyphal branching (Akiyama *et al*., 2005) as well as symbiosis with rhizobia in legumes in a complex concentration‐dependent manner (Foo & Davies, 2011; De Cuyper *et al*., 2014). Recent studies have shown that SLs also play a role in plant responses to various abiotic stresses. For instance, *Arabidopsis* mutants impaired in SL biosynthesis or signaling are hypersensitive to drought and salinity stress (Bu *et al*., 2014; Van Ha *et al*., 2014). Furthermore, SLs are also involved in plant defense responses to biotic stresses. For instance, the SL‐biosynthesis and sensing *max* mutants of *Arabidopsis* develop a stronger leafy gall syndrome than wild‐type (WT) controls do as a result of infection by the biotrophic actinomycete *Rhodococcus fascians* (Stes *et al*., 2015). Similarly, the SL‐deficient tomato (*Solanum lycopersicum*) *Slccd8* mutant showed an increased susceptibility to the necrotrophic foliar fungal pathogens *Botrytis cinerea* and *Alternaria alternata* (Torres‐Vera *et al*., 2014). This study also demonstrated that *Slccd8* mutants contained significantly lower amounts of salicylic acid (SA), jasmonic acid (JA), and abscisic acid (ABA) compared with the WT plants. In pea (*Pisum sativum*), the SL pathway did not show any effect on colonization by the soil‐borne oomycete *Pythium irregulare* (Blake *et al*., 2016).

The root‐knot nematode *Meloidogyne graminicola* is one of the most damaging plant parasitic nematodes infecting rice roots, especially in aerobic conditions (Bridge *et al*., 2005; De Waele & Elsen, 2007). It causes yield losses up to 32% (Bridge *et al*., 2005; De Waele & Elsen, 2007). *Meloidogyne graminicola* is an obligate biotrophic and sedentary root endoparasite. However, the infective second‐stage juveniles (J2s) that are vermiform in shape are migratory. Upon finding a suitable host plant, the J2s invade roots at the elongation zone and migrate through the root intercellular space until they reach the vascular cylinder to establish a feeding site, containing giant cells (Gheysen & Mitchum, 2011). The nematodes withdraw nutrients from these giant cells throughout their life cycle, which is completed within 2–3 wk. The hyperplasia and hypertrophy of the surrounding cells result in the formation of a root‐knot (gall) on the infected root (Bridge *et al*., 2005; Kyndt *et al*., 2014).

*Meloidogyne* spp. simultaneously influence plant cell differentiation and modulate the defense responses to establish a successful infection via feeding cell formation (Gheysen & Mitchum, 2011; Ji *et al*., 2013). The plant hormones JA, ethylene, SA, ABA, brassinosteroids (BR), gibberellic acid (GA, and auxin are known to be involved in the interactions between plant and root‐knot nematodes (Karczmarek *et al*., 2004; Nahar *et al*., 2011, 2013; Kyndt *et al*., 2017; Yimer *et al*., 2018; Gheysen & Mitchum, 2019). For instance, the JA‐based defense pathway plays a vital role in rice defense against *M. graminicola* infection, whereas SA is also involved in defense but to a lesser extent than JA (Nahar *et al*., 2011). In tomato, foliar application of methyl jasmonate (MeJA) and JA also induces defense against *M. incognita* and *M. javanica* infection in roots (Cooper *et al*., 2005). In addition, the JA‐precursor *cis*‐(+)‐12‐oxo‐phytodienoic acid (*cis*‐OPDA) acts as a signaling molecule in regulating *Arabidopsis* defense against *M. hapla* (Gleason *et al*., 2016). On the other hand, ABA, BR, and GA promote rice susceptibility to *M. graminicola* infection by interacting antagonistically with the JA pathway (Nahar *et al*., 2013; Kyndt *et al*., 2017; Yimer *et al*., 2018).

Here, we choose to study the interaction between rice as a monocot model plant and its economically important root pathogen *M. graminicola* to learn more about the function of SL in plant defense. First, we analyzed the expression of some genes related to the SL pathway and quantified defense‐related hormones in rice roots infected with *M. graminicola*. Upregulation of SL biosynthesis and signaling genes suggested a potential role of SL in rice–*M. graminicola* interaction. Then, we manipulated SL levels by treating plants with an SL analogue or an SL biosynthetic inhibitor before nematode inoculation, and analyzed the responses of rice *d* mutants that are deficient in SL biosynthesis and signaling. SL signaling was found to enhance *M. graminicola* infection and to decrease the levels of jasmonates.

## Materials and Methods

### Plant materials and growth conditions

Two SL‐biosynthetic rice *dwarf* (*d*) mutants (*d10* and *d27*), one SL‐signaling mutant *d14* and their corresponding WT (*O.  sativa* cv Shiokari) were used in this study. The seeds of these rice genotypes were provided by Professor Mikio Nakazono, Nagoya University, and Dr Itsuro Takamure, Hokkaido University, Japan. The seeds were first germinated in Petri dishes containing  moist tissue paper at 30°C for 4 d in the dark. Each germinated seedling was planted into a specially made polyvinylchloride tube containing sand and absorbent polymer (Reversat *et al*., 1999). Then, the seedlings were grown in a rice culture room under controlled environmental conditions (26°C : 24°C, day : night temperature, 70% relative humidity, 12 h : 12 h, light : dark cycle). Each plant was fertilized with 10 ml of Hoagland's solution two times per week.

### Nematode culture maintenance

The root‐knot nematode *M. graminicola* was provided by Professor Dirk De Waele (Catholic University Leuven, Belgium) and was originally isolated from rice in the Philippines. The nematode culture was multiplied and maintained using the susceptible rice cv Nipponbare or a grass‐host *Echinocloa crusgalli* in 2 l plastic pots having soil medium in similar growth conditions as mentioned earlier.

### Inoculation of rice seedlings with *M. graminicola* and susceptibility assessment

The J2s of *M. graminicola* were extracted from 3‐month‐old infected roots using the modified Baermann method (Luc *et al*., 2005). Then 2‐wk‐old rice seedlings were each inoculated with *c*. 200 J2s. The infected root samples were collected at 14 d post‐inoculation (dpi). To visualize the galls and the developmental stages of nematodes inside the galls, the nematode‐infected roots were boiled for 3 min in 0.8% acetic acid and 0.013% acid fuchsin solution. Then the roots were washed with running tap water and incubated in acidified glycerol. This treatment turns the galls and nematodes a purple color, while the rest of the root system is slowly destained in the acidified glycerol. First, the numbers of galls per plant were counted under a stereomicroscope (Leica S8 APO; Leica Microsystems, Wetzlar, Germany). Then each gall was dissected using a needle under the microscope. The developmental stages of all nematodes per plant were counted. The swollen female nematodes containing eggs were counted as egg‐laying females (ELFs), and this criterion was used as a measure of nematode development. The nematode infection experiments were performed at least twice, including at least six individual plants per treatment in each experiment.

### RNA isolation and complementary DNA synthesis

The WT rice plants (‘Shiokari’) were grown and infected by the root‐knot nematode *M. graminicola* as described previously. The whole root systems from both the infected and uninfected control plants were collected 1 d after inoculation. There were four biological replicates per treatment, and each biological replicate consisted of a pool of roots of four to six plants. The samples were immediately frozen in liquid nitrogen (N_2_) and ground to fine powder using a mortar and pestle. Total RNA was isolated from *c*. 100 mg finely ground root tissues using the Invitrap Spin Plant RNA Mini Kit (Stratec Biomedical, Birkenfeld, Germany) following the instructions of the manufacturer, except that an additional DNase treatment was included using a Qiagen RNase‐Free DNase Set as described by Ullah *et al*. (2017). The quantity and quality of the RNA was checked by spectrophotometry (NanoDrop 2000; Thermo Fisher Scientific, Wilmington, DE USA). Reverse transcription of *c*. 500 ng total RNA into complementary DNA (cDNA) was achieved by using SuperScript III reverse transcriptase (Invitrogen) and 50 pmol Oligo (dT)12‐18 Primer (Invitrogen) in a reaction volume of 20 μl. The cDNA was diluted five times with sterile water.

### Quantitative real‐time PCR

The quantitative PCR reactions were performed in a 20 μl volume containing 10 μl Brilliant III Ultra‐Fast SYBR Green QPCR Master Mix (Agilent Technologies, Santa Clara, CA, USA), 10 pmol forward and 10 pmol reverse primers, and 2 μl diluted cDNA (*c*. 50 ng). The quantitative real‐time (qRT)‐PCR was performed using a CFX Connect Real‐Time PCR Detection System (Bio‐Rad) using a two‐step amplification protocol (cycling parameters: 95°C for 3 min followed by 39 cycles of 95°C for 10 s and 58°C for 30 s). A melt‐curve analysis was performed for each sample run using the Bio‐Rad default parameters (95°C for 10 s, 65–95°C in 0.5°C increments) that yielded one peak. A nontemplate water sample was used as a negative control. Transcript abundance was calculated from five biological replicates for each treatment, with each biological sample consisting of three technical replicates, and was normalized to the abundance of the housekeeping gene *OsExp*. Relative expression was based on the $2^{-\Delta \Delta C_{T}}$ method (Livak & Schmittgen, 2001). The gene‐specific primers are provided in Supporting Information Table S1.

### Extraction of hormones from rice roots

Frozen rice roots were ground to fine powder in liquid N_2_. Tissues (*c*. 75 mg) were extracted with 1 ml analytical‐grade methanol containing 2 μl phytohormone standard mix (20 ng of D4‐SA, 20 ng of D6‐JA, and 4 ng D6‐JA–isoleucine (Ile) internal standards). The contents were vortexed vigorously for a few seconds, incubated at 20°C for 25 min at 1000 rpm, and centrifuged at 13 000 ***g*** at 4°C for 5 min. Supernatant (*c*. 950 μl) was transferred to a new microcentrifuge tube. The extracts were directly analyzed using LC–MS/MS for phytohormone quantification.

### Quantification of phytohormones by LC–MS/MS

For phytohormone analysis, chromatography was performed on an Agilent 1200 HPLC system (Agilent Technologies). An API 5000 tandem mass spectrometer (AB Sciex, Darmstadt, Germany) equipped with a turbo spray ion source was operated in negative ionization mode. Hormones were separated on a Zorbax Eclipse XDB‐C18 column (1.8 μm, 50 mm × 4.6 mm; Agilent) at 25°C, with two mobile phases consisting of 0.05% formic acid in water and acetonitrile. The flow rate and the elution profile were similar to the method described by Ullah *et al*. (2019). The parent ion and corresponding fragments of SA and the jasmonates were analyzed by multiple reaction monitoring as described by Vadassery *et al*. (2012). Mass data were collected and processed using analyst 1.6 software (Applied Biosystems). Linearity in ionization efficiencies was confirmed by analyzing serial dilutions of a standard mixture. The concentrations of *cis*‐OPDA, JA, JA–Ile, and SA were determined relative to the corresponding internal standard.

### Spraying with chemicals followed by nematode inoculation

The SL biosynthesis inhibitor TIS108 and the SL analogue GR24 were purchased from Chiralix (Nijmegen, the Netherlands). The JA biosynthesis inhibitor 5,8,11,14‐eicosatetraynoic acid (ETYA) was purchased from Sigma‐Aldrich. The TIS108 and ETYA were dissolved in ethanol, and GR24 was dissolved in acetone to make a 10 mM stock solution. The final spray concentrations were 3 μM for TIS108, 0.1 and 5 μM for GR24, and 100 μM for ETYA in distilled water containing 0.02% (v/v) Tween 20. Two‐week old rice seedlings were thoroughly sprayed until run‐off with these chemicals. For the mock‐treated plants, 0.02% (v/v) Tween 20 in distilled water containing 0.001% ethanol or acetone for TIS108 and ETYA or GR24 treatment was applied, respectively. Root samples were collected 24 h after spraying to analyze phytohormones. For each treatment, five biological replicates were used, each consisting of a pool with four to six plants. At the same time, 24 h after spraying, another eight plants per treatment were inoculated with *M. graminicola* to analyze the infection levels. Infected root samples were collected at 14 dpi and stained with acid fuchsin as described in the previous section.

### Statistical analysis

All data were analyzed by using the statistical package R v.3.2.0 (R Foundation for Statistical Computing, Vienna, Austria). Normality of data and homogeneity of variances were checked using the Shapiro–Wilk and Levene test, respectively. If test assumptions were not met, data were square root or log transformed. Data were then analyzed by one‐way ANOVA followed by Tukey's post‐hoc test to compare differences among the treatment groups. Comparisons between two means of different treatments were conducted using a two‐tailed Student's *t*‐test. Percentage data showing nematode development were analyzed using a chi‐squared test.

## Results

### *Meloidogyne graminicola* infection in rice activates the SL pathway genes, but inhibits jasmonate accumulation

**Figure 1:**
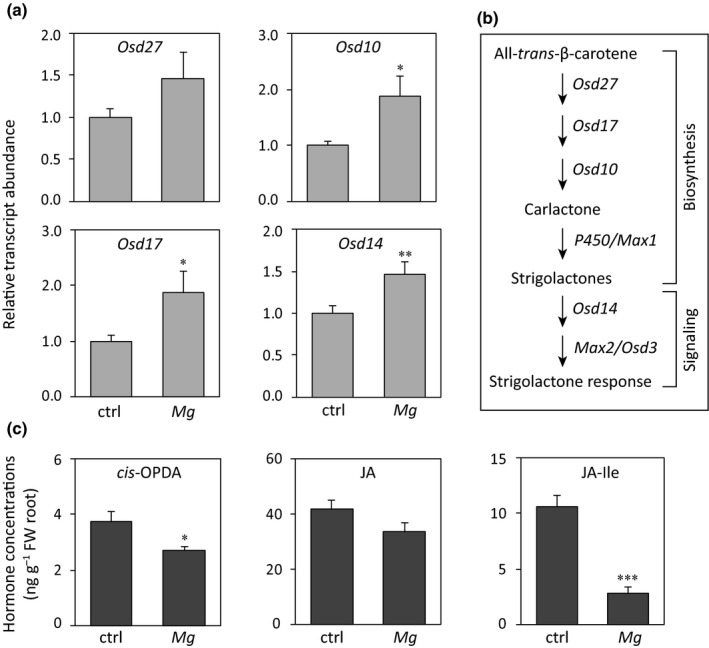
*Meloidogyne graminicola* infection in rice roots influences the strigolactone (SL) and jasmonic acid (JA) pathways in opposite ways. (a) Relative expression of SL biosynthesis and signaling genes with and without *M. graminicola* infection 1 d post‐inoculation (dpi). The messenger RNA levels were measured by quantitative real‐time PCR in three technical replicates per sample. Transcripts of each gene were normalized to *OsExp* transcripts. Data are presented as the mean + SE (*n* = 4), and each biological replicate was a pool of four to six plants. (b) Biosynthesis and signaling genes of SL pathway in rice. (c) Concentrations of jasmonates in rice roots with and without *M. graminicola* infection 1 dpi. Hormones were analyzed by LC–MS/MS. Data are presented as the mean + SE (*n* = 5), and each replicate was a pool of four to six plants. Data were analyzed using a two‐tailed Student's *t*‐test: *, *P *<* *0.05; **, *P *<* *0.01; and ***, *P *<* *0.001. ctrl, uninfected control; *Mg*,* M. graminicola* infected; *cis*‐OPDA,* cis*‐(+)‐12‐oxo‐phytodienoic acid; JA–Ile, JA–isoleucine.

To investigate the potential role of SL on root‐knot nematode infection in rice, the expression of SL biosynthesis and signaling genes were analyzed in rice roots 1 dpi with *M. graminicola* and compared with uninfected control roots by qRT‐PCR. Transcript levels of the first biosynthetic gene *Osd27* increased in nematode‐infected roots but were not significantly different from those in control roots (Fig. 1a). *Meloidogyne graminicola* infection in roots significantly induced the transcript levels of *Osd17* and *Osd10* compared with control roots (Fig. 1a). Furthermore, transcripts of the SL signaling gene *Osd14* (Fig. 1b) significantly increased in rice roots infected with *M. graminicola* compared with control roots (Fig. 1a). To determine whether defense hormones such as jasmonates and SA were changed during the early stage of the rice–*M. graminicola* interaction, their contents were quantified in infected and uninfected control roots. The levels of the JA precursor, *cis*‐OPDA, were significantly lower in roots infected by *M. graminicola* than in control roots (Fig. 1c). The level of JA decreased slightly upon nematode infection; however, the change was not statistically different in comparison with control roots. Interestingly, the level of the bioactive JA–Ile conjugate was three‐fold lower in roots infected by *M. graminicola* than in control roots (Fig. 1c). The contents of JA and the JA–Ile catabolites, sulfated‐JA (sulfo‐JA) and hydroxyl/carboxy‐JA–Ile (OH/COOH‐JA–Ile), respectively, increased in rice roots infected with *M. graminicola* compared with control roots (Fig. S1). The level of SA did not change due to nematode infection (Fig. S1). The content of ABA increased slightly in nematode‐infected roots, but was not significantly different from that of control roots (Fig. S1). These results suggest that *M. graminicola* infection in rice roots induces the SL pathway and suppresses the JA pathway.

### Foliar spraying with the SL analogue GR24 increases *M. graminicola* infection and reduces jasmonate accumulation, but these effects are reversed at high dose of GR24

To investigate the potential role of SL during root‐knot nematode infection in rice, rice shoots were treated with GR24, a functional SL analogue (Besserer *et al*., 2008). The shoots of WT rice plants were sprayed with two different concentrations of GR24. A subset of the treated plants was harvested to collect root samples 24 h after treatment for hormone analysis. Another subset of plants was inoculated with nematodes at 24 h after treatment. The plant responses to nematode infection were evaluated 14 dpi by counting the number of galls, nematodes, and their different development stages.

**Figure 2:**
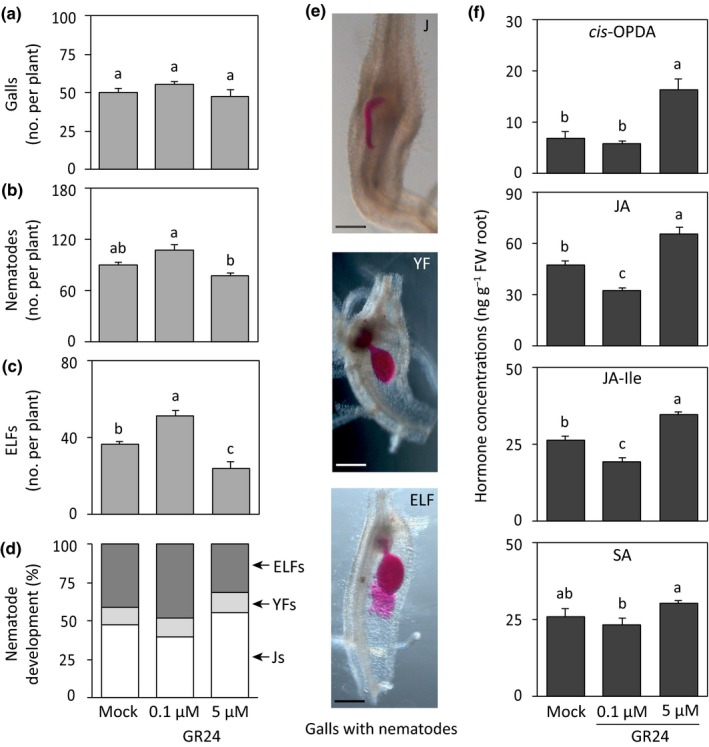
Effect of foliar spraying of the strigolactone analogue GR24 on *Meloidogyne graminicola* infection and the accumulation of phytohormones in rice roots. Fourteen‐day‐old wild‐type plants were sprayed with GR24 (0.1 or 5 μM) or with solvent only (mock). After 24 h, a subset of plants from each treatment were inoculated with *c*. 200 second‐stage juveniles of *M. graminicola* per plant and another subset of plants was sampled for hormone analysis. Plant responses were analyzed at 14 d post‐inoculation. Total numbers of (a) galls, (b) nematodes, (c) egg‐laying females (ELFs), and (d) percentages of the developmental stages of the observed nematodes within the galls per plant. (e) Representative pictures of the three developmental stages (J, juvenile; YF, young female; ELF) of nematodes inside galls observed under stereomicroscope after staining with acid fuchsin. (f) Concentrations of hormones were measured by LC–MS/MS in rice roots 24 h after spraying of GR24. Data were analyzed by one‐way ANOVA followed by Tukey's post‐hoc test. Different letters indicate statistically different means at 95% confidence. For nematode infection, data represent mean + SE (*n* = 6). For hormones, data represent mean + SE (*n* = 5), and each replicate was a pool of four to six plants. Percentage data shown in (d) were analyzed by a chi‐squared test (*P *=* *0.042). *cis*‐OPDA,* cis*‐(+)‐12‐oxo‐phytodienoic acid; JA, jasmonic acid; JA–Ile, JA–isoleucine; SA, salicylic acid. Bars, 1 mm.

Foliar application of GR24 did not affect the number of galls compared with mock‐treated plants (Fig. 2a). However, plants treated with 0.1 μM GR24 contained a higher total number of nematodes as well as ELFs per plant compared with the mock‐treated plants (Fig. 2b,c). By contrast, spraying with 5 μM GR24 decreased the total number of nematodes and ELFs per plant (Fig. 2b,c). The development of nematodes into ELFs increased by 7% in plants treated with 0.1 μM GR24, whereas there was a decrease by 10% in 5 μM GR24‐treated plants in comparison with mock treatment (Fig. 2d,e). Treatments of GR24 followed by nematode infection did not affect shoot and root lengths at 14 dpi compared with mock‐treated plants (Fig. S2).

The JA and SA pathways are known to play a pivotal role in rice defense against *M. graminicola* infection (Nahar *et al*., 2011; Kyndt *et al*., 2017). To investigate whether the contrasting effects of 0.1 and 5 μM GR24 on nematode development are also associated with jasmonates and SA, these defense hormones were analyzed in rice roots 24 h after foliar GR24 application. The content of *cis*‐OPDA was higher in roots of plants treated with 5 μM GR24 than in mock and 0.1 μM GR24‐treated plants (Fig. 2f). The levels of JA and JA–Ile both decreased significantly in 0.1 μM GR24‐treated plants, but their levels were significantly higher in plants treated with 5 μM GR24 than in mock‐treated plants (Fig. 2f). The contents of SA and ABA did not change in GR24‐treated plants compared with mock‐treated plants (Figs. 2f, S3). These results suggest that low doses of GR24 enhance *M. graminicola* infection and decrease JA accumulation in rice roots, but these effects are reversed at higher concentrations.

### SL deficiency in rice reduces the infection of *M. graminicola*

**Figure 3:**
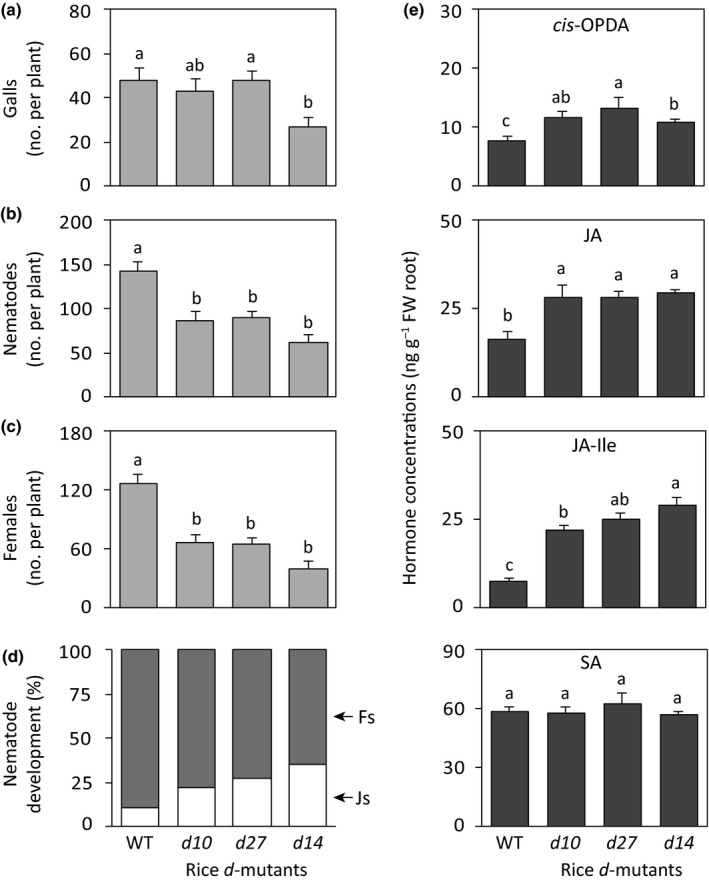
Responses of rice strigolactone (SL)‐biosynthesis and ‐signaling *dwarf* (*d*) mutants to *Meloidogyne graminicola* infection and phytohormone accumulation. The *d10* and *d27* lines are SL‐biosynthesis mutants, whereas *d14* is an SL‐signaling mutant. Each 14‐d‐old rice seedling was inoculated with *c*. 200 second‐stage juveniles of *M. graminicola*. Plant responses were analyzed 14 d post‐inoculation. Reported are the total number of (a) galls, (b) nematodes, (c) egg‐laying females, and (d) percentages of the developmental stages of the observed nematodes within the galls of each plant. (e) Enhanced accumulation of jasmonates in rice *d* mutants. The roots of 14‐d‐old rice seedlings were harvested before nematode inoculation, and hormone metabolites were analyzed by LC–MS/MS. Data were analyzed by one‐way ANOVA followed by Tukey's post‐hoc test. Different letters indicate statistically different means at 95% confidence. For the infection experiment, data represent mean + SE (*n* = 8). For hormone measurement, data represent mean + SE (*n* = 5) and each replicate was a pool of four to six plants. Percentage data shown in (d) were analyzed by a chi‐squared test (*P *<* *0.001). Fs, females (young females + egg‐laying females); Js, juveniles; *cis*‐OPDA,* cis*‐(+)‐12‐oxo‐phytodienoic acid; JA, jasmonic acid; JA–Ile, JA–isoleucine; SA, salicylic acid.

Next, the effect of reduced SL on the rice–*M. graminicola* interaction was investigated by inoculating the nematodes into two SL‐biosynthetic rice *dwarf* (*d*) mutants, *d10* and *d27*, one SL‐signaling mutant *d14* along with their respective WT plants. The nematode infection levels were analyzed by counting the number of galls per plant and the total number of nematodes, including their developmental stages at 14 dpi. The number of galls per plant was similar in *d10*,* d27*, and WT plants but was significantly lower in the SL‐signaling *d14* mutant (Fig. 3a). Interestingly, the total number of nematodes per plant and females, both young females and ELFs, were significantly lower in all *d* mutants than in WT plants (Fig. 3b,c). Whereas *c*. 90% of total nematodes developed into females in WT plants, *c*. 65–78% of the nematodes developed in the *d* mutants (Fig. 3d). The difference in the total number of nematodes and females per plant was related to the difference in gall size between *d* mutants and WT plants (Fig. S4). In the *d* mutants, only 3–5% galls were (extra‐)large, whereas *c*. 20% galls were (extra‐)large in the WT plants. The large and extra‐large galls were full with many more developed females and egg masses than the small and medium‐sized galls (Fig. S4). These results indicate that decreased SL levels in rice reduce infection by the root‐knot nematode *M. graminicola*.

Plant susceptibility to root‐knot nematode infection might be influenced by the root architecture, especially by the number of root tips, which is depicted by the number of lateral roots. Because the root tips are the entry points of this nematode (Gheysen & Mitchum, 2011), fewer root tips could lead to lower nematode infection. To investigate if root architecture has an influence on the lower nematode susceptibility in the *d* mutants, the root morphology of the *d* mutants and WT plants was analyzed. Total root length per plant was lower in all *d* mutants, but only significantly different in *d10* and *d14* compared with the WT plants (Fig. S5a). The *d* mutants also had significantly lower root surface area than WT plants did (Fig. S5b). However, the number of root tips, represented by the number of lateral roots, was comparable in WT and *d* mutant plants except for *d10* (Fig. S5c). The lateral root density was significantly higher in the SL‐signaling *d14* mutant than in the WT plants (Fig. S5d). These results suggest that the lower susceptibility of rice *d* mutants to *M. graminicola* infection is not associated with the root phenotypes.

### SL biosynthesis and signaling rice mutants contain higher jasmonate levels

To investigate whether the lower *M. graminicola* susceptibility in SL mutants was due to altered JA‐ and SA‐based defense pathways in rice, these hormone metabolites were quantified in roots of SL‐deficient and ‐signaling *d* mutants and compared with the amounts in WT plants. The level of the JA precursor, *cis*‐OPDA, JA, and its conjugate JA–Ile were higher in all *d* mutants than in the WT plants. The concentrations of JA and JA–Ile were up to two‐ and four‐fold higher, respectively, in *d* mutants in comparison with WT plants. On the other hand, the levels of SA and ABA in *d* mutants were similar to those in WT plants (Figs 3e, S3). These results suggest that SL deficiency induces the accumulation of jasmonates in rice roots.

### GR24 treatment restores *M. graminicola* susceptibility in SL‐deficient biosynthesis mutants in rice, but not in a signaling mutant

**Figure 4:**
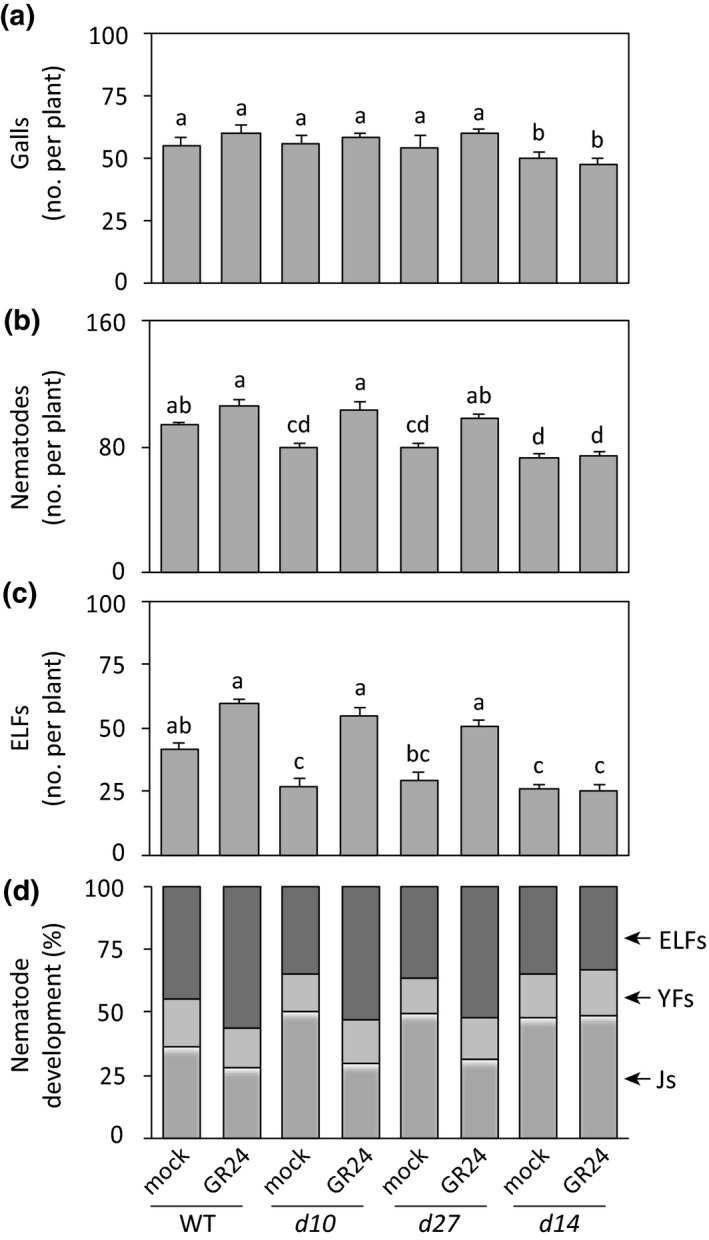
Foliar application of the strigolactone (SL) analogue GR24 restores susceptibility to *Meloidogyne graminicola* in SL‐deficient biosynthesis mutants (*d10* and *d27*), but not in the SL‐signaling mutant *d14*. Fourteen‐day‐old wild‐type (WT) and mutant plants were sprayed on the shoots with 0.1 μM GR24 and the respective mock plants were sprayed with solvent only. After 24 h, each plant was inoculated with *c*. 200 second‐stage juveniles of *M. graminicola*. Infection levels were analyzed at 14 d post‐inoculation by counting the total number of (a) galls, (b) nematodes, (c) egg‐laying females (ELFs), and (d) percentages of the developmental stages of the observed nematodes within the galls per plant. Data were analyzed by one‐way ANOVA followed by Tukey's post‐hoc test. Different letters indicate statistically different means at 95% confidence. Data represent mean + SE (*n* = 6). Percentage data (mock vs GR24) for each genotype shown in (d) were analyzed by a chi‐squared test (WT,* P *=* *0.09; *d10*,* P *=* *0.018; *d27*,* P *=* *0.04; *d10*,* P *=* *0.97). YFs, young females; Js, juveniles.

A complementation experiment was performed by treating the SL‐deficient *d* mutants and WT plants with GR24 at a concentration (0.1 μM) that promotes *M. graminicola* infection. Both GR24 and mock‐treated plants were inoculated with *M. graminicola* 24 h after treatment. The number of galls per plant at 14 dpi did not differ due to GR24 treatment compared to the corresponding mock‐treated plants (Fig. 4a). However, GR24 treatment significantly increased the total number of nematodes per plant in SL‐deficient *d10* and *d27* mutants (Fig. 4b). On the other hand, *M. graminicola* susceptibility did not increase due to GR24 treatment in *d14* plants, which are insensitive to SL (Fig. 4b). The development of nematodes into ELFs was restored in GR24‐treated SL biosynthetic *d* mutants (*d27* and *d10*) to wild‐type levels, but not in the SL signaling *d14* mutant (Fig. 4c,d). The shoot lengths of all rice *d*‐mutants were significantly lower than corresponding wild‐type plants, irrespective of GR24 and nematode inoculation (Fig. S6). However, the root lengths were similar in all plants at 14 dpi (Fig. S6). This experiment confirmed the role of SL signaling in promoting *M. graminicola* susceptibility.

To verify the role of GR24 in suppressing the jasmonate pathway, we conducted another experiment using the SL‐biosynthetic mutants (*d10* and *d27*) and SL‐signaling mutant (*d14*). Two‐week‐old seedlings were sprayed with GR24 (0.1 μM) and or only solvent (mock). Hormones were measured in whole‐root tissues at 1 d after spraying. In general, the levels of *cis*‐OPDA, JA and JA‐Ile decreased in SL‐biosynthetic mutants after GR24 application (Fig. S7A). This declining trend was significantly different in *d10* (JA, JA‐Ile), *d27* (JA‐Ile) mutants when compared between mock and GR24 treated plants. By contrast, the levels of sulfo‐JA, OH‐JA, and COOH‐JA‐Ile slightly increased after GR24 treatment (Fig. S7B). Interestingly, the levels of JA, JA‐Ile and other jasmonates were not reduced after GR24 treatment in the *d14* mutant, which is insensitive to SL (Fig. S7). Moreover, the levels of SA and ABA did not change in all *d*‐mutants after treatment with GR24 (Fig. S7).

### Foliar application of the SL biosynthesis inhibitor TIS108 suppresses *M. graminicola* infection and enhances the accumulation of jasmonates in rice roots

**Figure 5:**
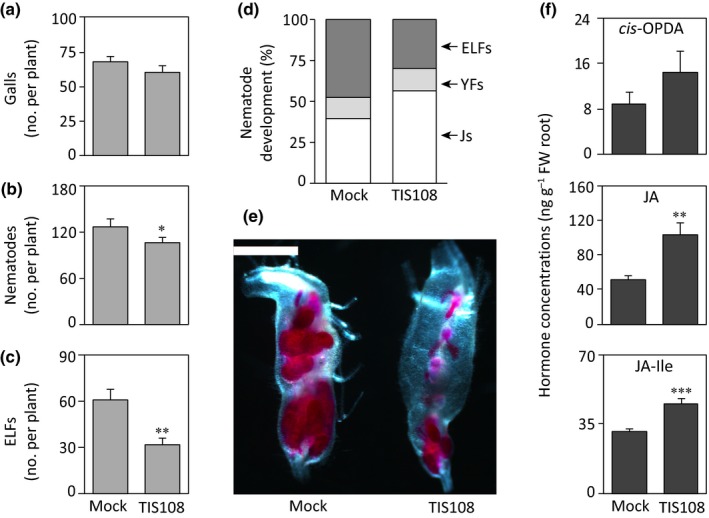
Foliar spraying of the strigolactone (SL) biosynthesis inhibitor TIS108 decreased *Meloidogyne graminicola* infection in rice roots and increased the contents of jasmonates. The shoots of 14‐d‐old wild‐type plants were sprayed with TIS108 (3 μM) or with solvent only (mock). After 24 h, a subset of plants from each treatment were inoculated with *c*. 200 second‐stage juveniles of *M. graminicola* per plant and another subset of plants were sampled for hormone analysis. Nematode infection levels were analyzed at 14 d post‐infection. Total numbers of (a) galls, (b) nematodes, (c) egg‐laying females (ELFs), and (d) percentages of the developmental stages inside the galls per plant. (e) A representative picture of galls in mock‐ and TIS108‐treated plants observed under a stereomicroscope after staining with acid fuchsin. (f) Concentrations of jasmonates were measured by LC–MS/MS in rice roots 24 h after spraying of TIS108. Data were analyzed by a two‐tailed Student's *t*‐test (*, *P *≤* *0.05; **, *P *≤* *0.01; ***, *P *≤* *0.001. For nematode infection, data represent mean + SE (*n* = 8). For hormone measurements, data represent mean + SE (*n* = 5), and each replicate was a pool of four to six plants. Percentage data shown in (d) were analyzed by a chi‐squared test (*P *=* *0.015). YFs, young females; Js, juveniles; *cis*‐OPDA,* cis*‐(+)‐12‐oxo‐phytodienoic acid; JA, jasmonic acid; JA–Ile, JA–isoleucine. Bar, 1 mm.

To confirm the ability of SL deficiency in rice to cause lower susceptibility to root‐knot nematode infection and upregulation of the JA pathway, the shoots of 2‐wk‐old WT plants were sprayed with TIS108, an inhibitor of SL biosynthesis (Ito *et al*., 2011). At 24 h after spraying the plants were inoculated with *M. graminicola*. The TIS108 treatment did not change the number of galls (Fig. 5a), however, it significantly reduced the total number of nematodes (Fig. 5b) and ELFs (Fig. 5c) per plant compared with the mock‐treated plants at 14 dpi. Approx. 50% of nematodes developed into ELFs in mock‐treated plants at 14 dpi, whereas only 30% developed into this form in TIS108‐treated plants (Fig. 5d). Moreover, the galls in mock‐treated plants contained a higher number of ELFs, whereas a higher number of juveniles was found in the galls in TIS108‐treated plants (Fig. 5e). The average lengths of the shoots and roots were similar in TIS108‐ or mock‐treated plants followed by *M. graminicola* infection at 14 dpi (Fig. S8). To investigate whether foliar treatment with TIS108 also affects the accumulation of jasmonates, the levels of jasmonates in roots were analyzed 24 h after treatment. While the level of *cis*‐OPDA increased slightly in TIS108‐treated plants (Fig. 5f), both JA and JA–Ile concentrations increased significantly by approximately two‐fold in roots of the TIS108‐treated plants compared with the mock‐treated plants (Fig. 5b,c). However, the levels of SA and ABA did not change due to the inhibition of the SL biosynthesis by TIS108 (Fig. S9).

### Reduced *M. graminicola* infection due to SL deficiency in rice is dependent on the jasmonate pathway

**Figure 6:**
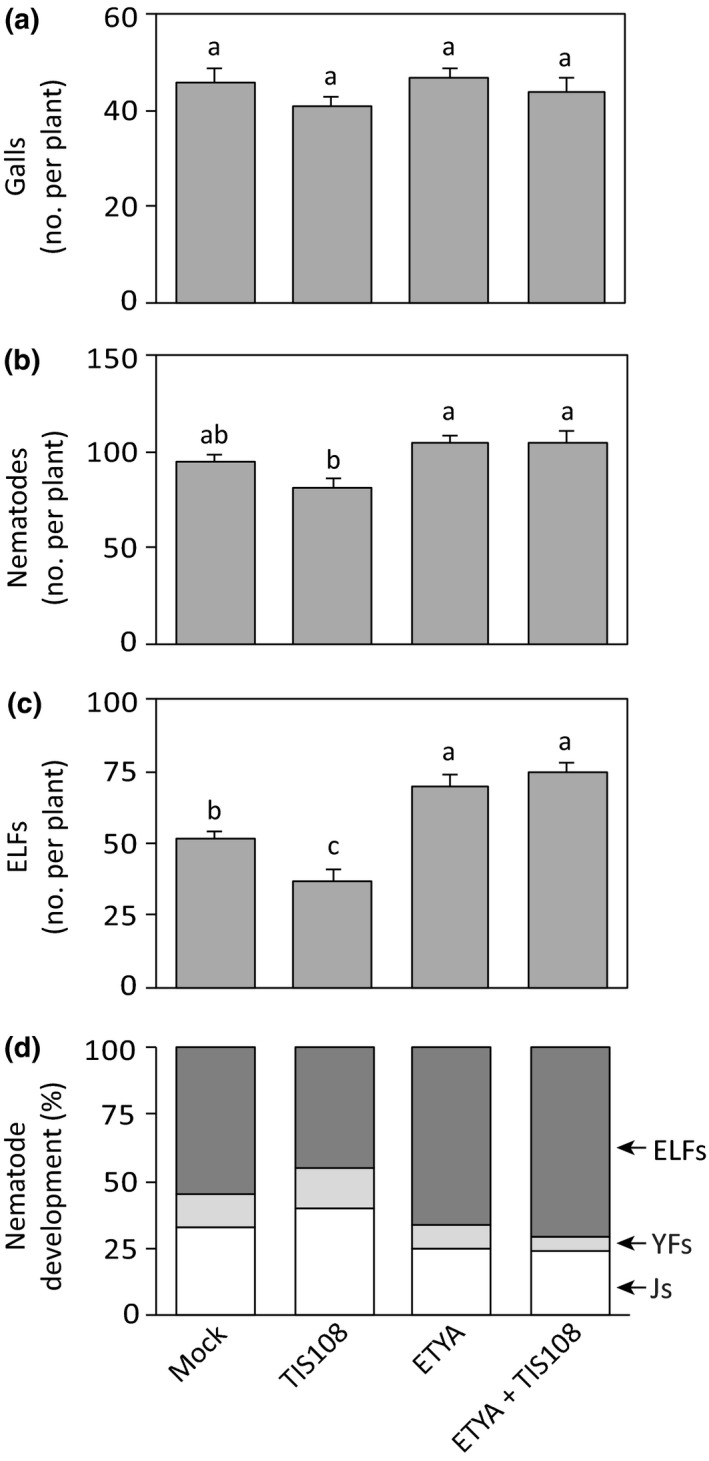
Effect of the strigolactone biosynthesis inhibitor TIS108 and the jasmonic acid (JA) biosynthesis inhibitor 5,8,11,14‐eicosatetraynoic acid (ETYA) on nematode infection. Inhibitors were sprayed on the shoots alone or in combination 24 h before *Meloidogyne graminicola* inoculation. Nematode infection was assessed 14 d post‐infection by counting total number of (a) galls, (b) nematodes, (c) egg‐laying females (ELFs), and (d) percentages of the developmental stages of the observed nematodes within the galls per plant. Data were analyzed by one‐way ANOVA followed by Tukey's post‐hoc test. Different letters indicate means were statistically different at 95% confidence. Data represent mean + SE (*n* = 6). Percentage data shown in (d) were analyzed by a chi‐squared test (*P *=* *0.01). YFs, young females; Js, juveniles.

To investigate whether the resistance to *M. graminicola* infection in rice arising from SL deficiency is dependent on the JA pathway, WT plants were treated with the SL biosynthetic inhibitor TIS108, and the JA biosynthetic inhibitor ETYA, which inhibits the lipoxygenase enzyme (Taheri & Tarighi, 2010). The foliar application of TIS108 or ETYA alone as well as their co‐application 24 h before nematode inoculation did not change the number of galls per plant (Fig. 6a). However, as seen before (Fig. 5b,c), the application of TIS108 alone decreased the total number of nematodes and ELFs per plant compared with the mock‐treated plants (Fig. 6b,c). By contrast, foliar application of ETYA significantly increased the total number of nematodes and ELFs per plant (Fig. 6b,c), consistent with the importance of JA in rice defense against this nematode (Nahar *et al*., 2011). Interestingly, the co‐application of TIS108 and ETYA also increased susceptibility to *M. graminicola* infection to the same level as ETYA treatment alone (Fig. 6). These data suggest that the suppression of *M. graminicola* upon SL deficiency infection in rice depends on jasmonate biosynthesis.

## Discussion

SLs, a relatively recently described group of plant hormones, have been extensively studied in the last decade. These hormones are known to play an important role in various aspects of plant developmental and physiological processes, including shoot branching (Gomez‐Roldan *et al*., 2008; Umehara *et al*., 2008), leaf senescence (Ueda & Kusaba, 2015), and root development (Kapulnik *et al*., 2011; Ruyter‐Spira *et al*., 2011; Arite *et al*., 2012). They also stimulate seed germination of root‐parasitic weeds (Cook *et al*., 1966), promote arbuscular mycorrhizal association with their host plants (Akiyama *et al*., 2005), and affect nodulation in legumes in a dose‐dependent manner (De Cuyper *et al*., 2014). A few studies also demonstrated that SLs play a role in plant responses to abiotic stress; for example, drought tolerance (Bu *et al*., 2014; Van Ha *et al*., 2014), salt tolerance (Van Ha *et al*., 2014), and root development under nutrient deficiencies (Sun *et al*., 2014). Whether SLs play a role in plant–pathogen interactions is still under investigation. The rice root‐knot nematode *M. graminicola* causes substantial yield losses in rice production, especially under aerobic conditions. This nematode is able to manipulate developmental processes in the host cell and suppress innate immunity during infection (Kyndt *et al*., 2012). Our previous work described the importance of plant hormones such as JA, SA, ABA, ethylene, BR and auxin during the rice–*M. graminicola* interaction (Nahar *et al*., 2011, 2013; Kyndt *et al*., 2017; Yimer *et al*., 2018). Here, we focus on the role of SLs in the interaction between rice and *M. graminicola*.

In our study, *M. graminicola* infection in rice roots transcriptionally activated SL biosynthesis and signaling at 1 d after inoculation, a time when root‐knot nematodes initiate feeding site induction. We also measured defense hormones and found that the jasmonate levels were strongly reduced in nematode‐infected roots. Accumulation of hydroxylated‐ and carboxylated‐JA–Ile, which seem to be inactive forms (Wasternack & Strnad, 2018), in nematode‐infected roots also correlated with the reduction of bioactive JA–Ile. Upregulation of the SL signaling pathway and suppression of the JA pathway raised the question of whether SLs play a direct role in rice defense against *M. graminicola* infection. To address this question, SL biosynthesis and signaling mutants were analyzed in a nematode infection experiment. The infection and development of *M. graminicola* were significantly lower in SL‐deficient (*d27* and *d10*) and ‐responsive (*d14*) rice *d* mutants, suggesting that SLs act as negative regulators of rice defense against root‐knot nematode infection. Inhibition of SL biosynthesis by spraying TIS108 also resulted in lower nematode susceptibility in rice roots. In contrast to our result, a previous study reported an increased susceptibility to the necrotrophic foliar fungi *B. cinerea* and *A. alternata* in the SL‐deficient tomato *Slccd8* mutant (equivalent to the *d10* rice mutant) (Torres‐Vera *et al*., 2014). Similarly, in *Arabidopsis*, SL deficiency promoted plant susceptibility to the biotrophic actinomycete *R. fascians*, causing leafy gall syndrome (Stes *et al*., 2015). However, the SL pathway did not show any effect on pea defense during the soil‐borne oomycete *P. irregulare* infection (Blake *et al*., 2016). Therefore, the role of SLs in plant defense against pathogen infections appears to vary in different plants, different tissues, and with different types of attackers.

On the other hand, treatment with a low dose of GR24 (a functional analogue of SL; Besserer *et al*., 2008) on rice shoots 24 h before nematode inoculation increased susceptibility to *M. graminicola* infection. A foliar spray with 0.1 μM GR24 significantly increased the number of ELFs at 14 dpi, but treatment with 5 μM GR24 decreased infection levels. In agreement with our observations, the number of nodules increased in *Medicago truncatula* treated with 0.1 μM GR24, whereas they were strongly reduced after treatment with 5 μM GR24 (De Cuyper *et al*., 2014). Application of GR24 at low concentration restored the nematode susceptibility in the SL‐biosynthesis mutants (*d27* and *d10*), but not in the SL signaling mutant (*d14*), which is deficient in SL/GR24 perception. These findings suggest that SL signaling in rice is required for enhanced root‐knot nematode susceptibility. In rice, phosphate and nitrate deficiency‐induced reduction in lateral root density was rescued by GR24 treatment in WT as well as in SL‐biosynthesis mutants but not in the SL‐signaling mutant (Sun *et al*., 2014). SL signaling plays an important role in determining rice root architecture. Thus, one could argue that SL deficiency in the *d* mutants might have negatively influenced nematode infection levels by decreasing lateral root density, as the root tips are the sites of *M. graminicola* penetration into the host. However, we found a higher lateral root density in SL‐deficient and ‐signaling *d* mutants, suggesting that the possibility of nematode host entrance is not decreased in these mutant lines even though these plants were less susceptible to *M. graminicola* infection.

Since the root phenotypes of rice *d* mutants do not seem to be at the basis of their increased *M. graminicola* resistance, we hypothesized that altered defense hormones might be involved. Plant hormones, especially JA‐mediated defense signaling, play an important role in both monocot and dicot plant species to protect against root‐knot nematode infections (Cooper *et al*., 2005; Nahar *et al*., 2011; Gheysen & Mitchum, 2019). It was shown that the JA‐precursor *cis*‐OPDA also acts as a key signaling molecule in the regulation of *Arabidopsis* defense against the root‐knot nematode *M. hapla* (Gleason *et al*., 2016). On the other hand, SA signaling also mediates defense in rice against *M. graminicola* infection, but to a lesser extent than JA (Nahar *et al*., 2011).

In this study, we could show a negative association between active SL biosynthesis and signaling and JA accumulation in rice roots. The contents of *cis*‐OPDA, JA, and JA–Ile all significantly increased in the roots of SL biosynthesis and signaling rice *d* mutants in comparison with the WT plants. In line with these results, chemical inhibition of SL biosynthesis by TIS108 (Ito *et al*., 2011) also increased the levels of jasmonates in rice roots, whereas no effects on SA were observed. Therefore, decreased root‐knot nematode susceptibility in SL‐deficient rice mutants appears to be due to the activation of the JA pathway, which is known as a positive regulator of rice defense against *M*. *graminicola* infection (Nahar *et al*., 2011). In contrast to our results, the tomato *Slccd8* mutant showed reduced levels of defense hormones, including JA and SA, which consequently increased susceptibility to the foliar necrotrophic fungi *B. cinerea* and *A. alternata* (Torres‐Vera *et al*., 2014). However, JA accumulation was not affected in SL‐deficient (*max1*) or ‐responsive mutant (*max2*) of *Arabidopsis thaliana* (Rozpądek *et al*., 2018). By contrast, the *max1* mutant accumulated a higher amount of SA than WT plants did. In *Arabidopsis*, GR24 treatment at 1 μM increased the production of SA in roots (Rozpądek *et al*., 2018). However, GR24 treatment in rice did not change the contents of SA in our study. It is possible that the interplay between SL and defense hormones such as JA and SA is different in monocot and dicot plants, or that their interaction is concentration dependent. Indeed, in our study, the levels of JA and JA–Ile decreased in systemic roots treated with 0.1 μM GR24 on the foliage, but increased after treatment with 5 μM GR24, and nematode infection correlated inversely with the jasmonate level. Furthermore, we could show that foliar application of GR24 on *d10* and *d27* lines (SL‐biosynthetic mutants) reduced the levels of JA and JA–Ile but not in *d14* signaling mutant that is unable to perceive exogenous SL or GR24. These findings indicate that increased root‐knot nematode susceptibility after GR24 application in SL‐biosynthetic mutants might be due the suppression of active JA signaling. In addition, we showed that chemical inhibition of the JA pathway by ETYA increased *M. graminicola* susceptibility in rice, whereas treatment with the SL biosynthetic inhibitor TIS108 decreased susceptibility. The co‐application of both of these inhibitors resulted in nematode susceptibility to a similar level as ETYA treatment alone, suggesting that increased nematode resistance due to SL deficiency is dependent on an intact JA pathway.

Because ABA and SLs share the same biosynthetic precursor, β‐carotene, we also investigated whether ABA plays a role in SL‐induced *M. graminicola* susceptibility. It is known that ABA plays a complex role in the interaction between rice and *M. graminicola* (Kyndt *et al*., 2017). In our study, neither the exogenous GR24 treatment nor the TIS108 treatment or the *d*‐mutants changed the contents of ABA in rice roots. In agreement with our findings, chemical inhibition of SL biosynthesis in tomato did not affect the ABA content in roots (López‐Ráez *et al*., 2010). However, this study also proposed that endogenous ABA might be important for SL synthesis because inhibition of the ABA pathway compromised SL production. Our findings do not support any direct relation between SLs and ABA in rice roots.

**Figure 7:**
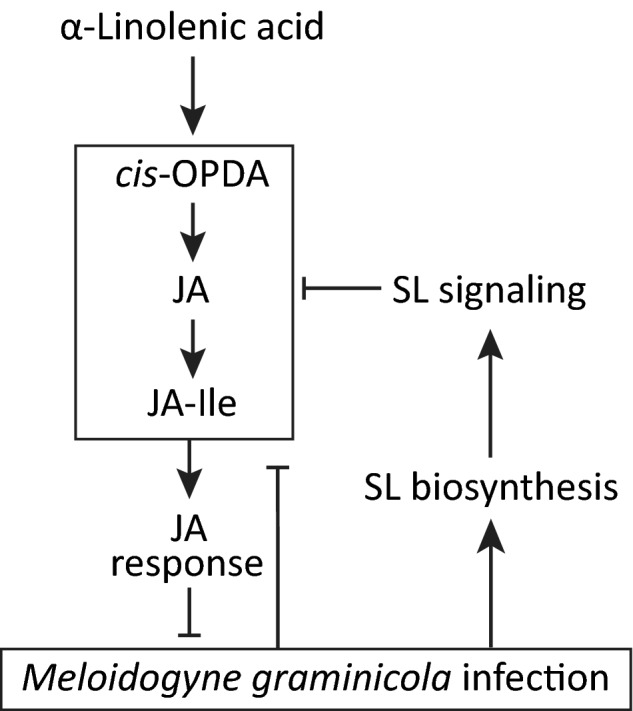
A model depicting the antagonism between the strigolactone (SL) and the jasmonic acid (JA) pathway in rice roots. *Meloidogyne graminicola* infection in rice roots induces the SL pathway and represses the JA pathway. On the other hand, the JA pathway reduces *M. graminicola* infection and the SL pathway represses the JA pathway to promote infection. *cis*‐OPDA,* cis*‐(+)‐12‐oxo‐phytodienoic acid; JA–Ile, JA–isoleucine.

In conclusion, this study shows that SLs play an important role in the interaction between rice and the root‐knot nematode *M. graminicola*. Inhibition of SL biosynthesis in rice either chemically or genetically resulted in enhanced JA accumulation, which led to a lower nematode infection. During the early stage of *M. graminicola* infection in rice roots, SL biosynthesis is increased, and this is also associated with a strong downregulation of JA‐induced defense response. We conclude that SLs enhance susceptibility of rice to *M. graminicola* by antagonizing the JA biosynthesis pathway (Fig. 7).

## Author contributions

ZL, CU, TK and GG designed the study. ZL and CU carried out experiments and analyzed data. ZL prepared the draft manuscript. CU, TK, JG and GG helped in critical discussion and revised the manuscript.

## Supporting information

Please note: Wiley Blackwell are not responsible for the content or functionality of any Supporting Information supplied by the authors. Any queries (other than missing material) should be directed to the *New Phytologist* Central Office.

**Fig. S1** Changes in salicylic acid, abscisic acid and JA‐catabolites in rice roots upon *Meloidogyne graminicola* infection.
**Fig. S2** Effect of foliar GR24 application followed by *M. graminicola* infection on shoot and root lengths of rice plants.
**Fig. S3** Abscisic acid concentrations in rice roots after foliar application of GR24 and in rice *d*‐mutants.
**Fig. S4** Percentages of *M. graminicola* induced galls of different sizes in rice *d* mutants.
**Fig. S5** Root system architectures of rice *d*‐mutants and the corresponding wild‐type plants.
**Fig. S6** Effect of foliar GR24 application followed by *M. graminicola* infection on shoot and root lengths of rice *d*‐mutants and wild‐type plants.
**Fig. S7** Hormone metabolites in roots of the rice *d*‐mutants after GR24 application.
**Fig. S8** Effect of foliar TIS108 application followed by *M. graminicola* infection on shoot and root lengths of rice plants.
**Fig. S9** Effect of foliar application of strigolactone biosynthesis inhibitor (TIS108) on salicylic acid and abscisic acid contents.
**Table S1** Primer sequences used in this study for qRT‐PCR.
