# Supplementary material for: Strigolactones enhance root‐knot nematode (Meloidogyne graminicola) infection in rice by antagonizing the jasmonate pathway
Source: New Phytol. 2019 Jul 2;224(1):454–65. doi: 10.1111/nph.15953 (PMC6852604; doi:10.1111/nph.15953)
Supplement: Supplementary file 1 — Fig. S1 Changes in salicylic acid, abscisic acid and JA‐catabolites in rice roots upon Meloidogyne graminicola infection. Fig. S2 Effect of foliar GR24 application followed by M. graminicola infection on shoot and root lengths of rice plants. Fig. S3 Abscisic acid concentrations in rice roots after foliar application of GR24 and in rice d‐mutants. Fig. S4 Percentages of M. graminicola induced galls of different sizes in rice d mutants. Fig. S5 Root system architectures of rice d‐mutants and the corresponding wild‐type plants. Fig. S6 Effect of foliar GR24 application followed by M. graminicola infection on shoot and root lengths of rice d‐mutants and wild‐type plants. Fig. S7 Hormone metabolites in roots of the rice d‐mutants after GR24 application. Fig. S8 Effect of foliar TIS108 application followed by M. graminicola infection on shoot and root lengths of rice plants. Fig. S9 Effect of foliar application of strigolactone biosynthesis inhibitor (TIS108) on salicylic acid and abscisic acid contents. Table S1 Primer sequences used in this study for qRT‐PCR. [file NPH-224-454-s001.pdf]

New Phytologist

New Phytologist Supporting Information

Article title: Strigolactones enhance root-knot nematode (*Meloidogyne graminicola*) infection in rice by antagonizing the jasmonate pathway

Authors: Zobaida Lahari, Chhana Ullah, Tina Kyndt, Jonathan Gershenzon, Godelieve Gheysen

Article acceptance date: 16 May 2019

The following Supporting Information is available for this article:

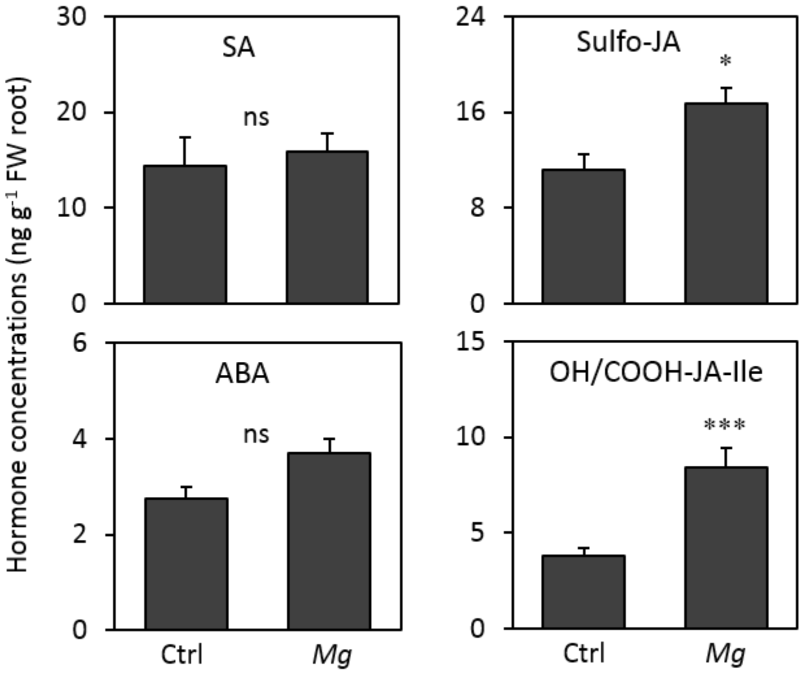

Fig. S1 Changes in salicylic acid, abscisic acid and JA-catabolites in rice roots upon *Meloidogyne graminicola* infection. Hormone metabolites were measured with and without nematode infection at 1 day post inoculation. Data were analyzed using a two-tailed Student's t-test. *, $p < 0.05$; and ***, $p < 0.001$; ns = not significant. ctrl = uninfected control, *Mg* = *M. graminicola* infected. OH/COOH-JA-Ile = 12-Hydroxy/-Carboxy-JA-Ile. Data represent mean + SE (n=5), and each replicate was a pool of 4-6 plants.

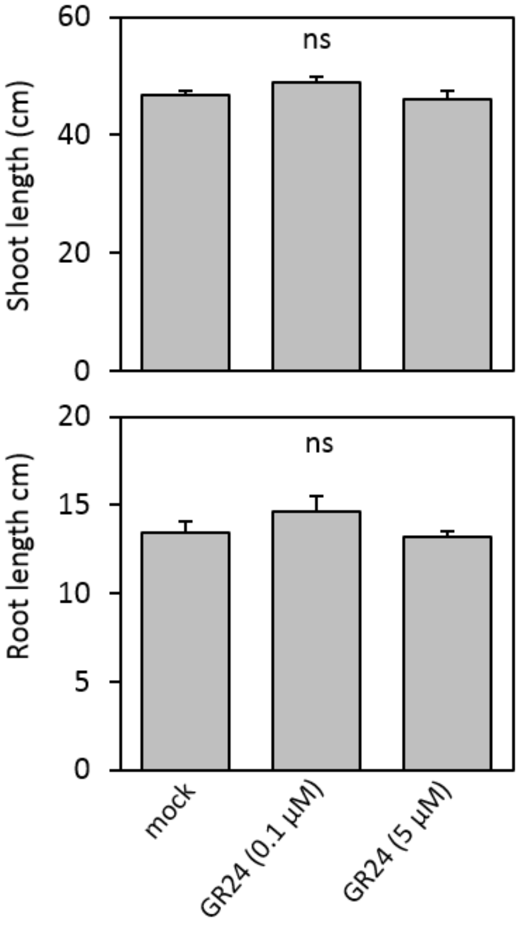

Fig. S2 Effect of foliar GR24 application followed by *Meloidogyne graminicola* infection on shoot and root lengths of rice plants. Two-week-old wild type rice seedlings were sprayed with GR24 (0.1 µM or 5 µM) and mock plants were sprayed with solvent only. After 24 hours, each plant was inoculated with around 200 second stage juveniles. Shoot and root length were measured at 14 dpi. Data were analyzed by one-way ANOVA (ns = not significant). Data represent mean + SE (n=6).

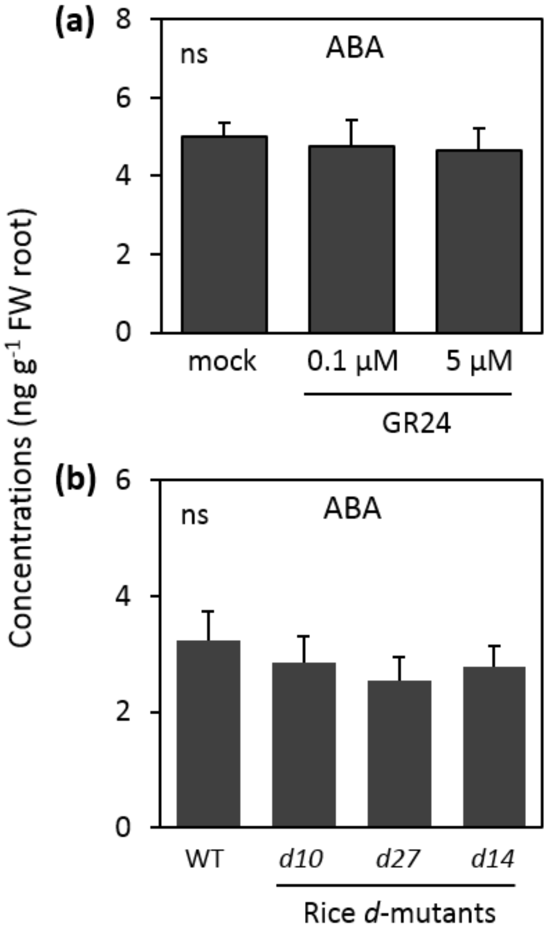

Fig. S3 Abscisic acid concentrations in rice roots after foliar application of GR24 and in rice *d*-mutants. **(a)** ABA contents in rice roots after GR24 treatment. Two-week-old wild type rice seedlings were sprayed with GR24 and their respective mock plants were sprayed with solvent only. After 24 hours, roots were harvested. **(b)** ABA contents in rice *d*-mutants. The *d10* and *d27* are strigolactone biosynthesis mutants and *d14* is signaling mutant. The roots of 14-day-old rice seedlings were harvested before nematode inoculation and hormone metabolites were measured using LC-tandem mass sprectrometry. Data were analyzed by one-way ANOVA (ns = not significant). Data represent mean + SE (n=5).

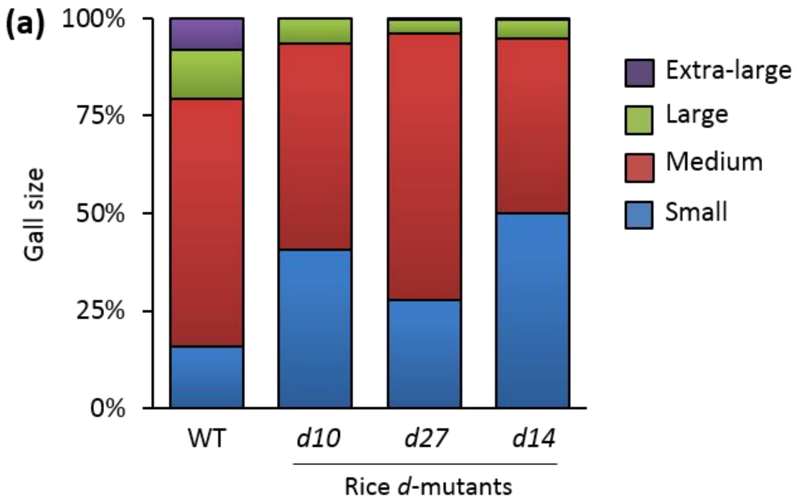

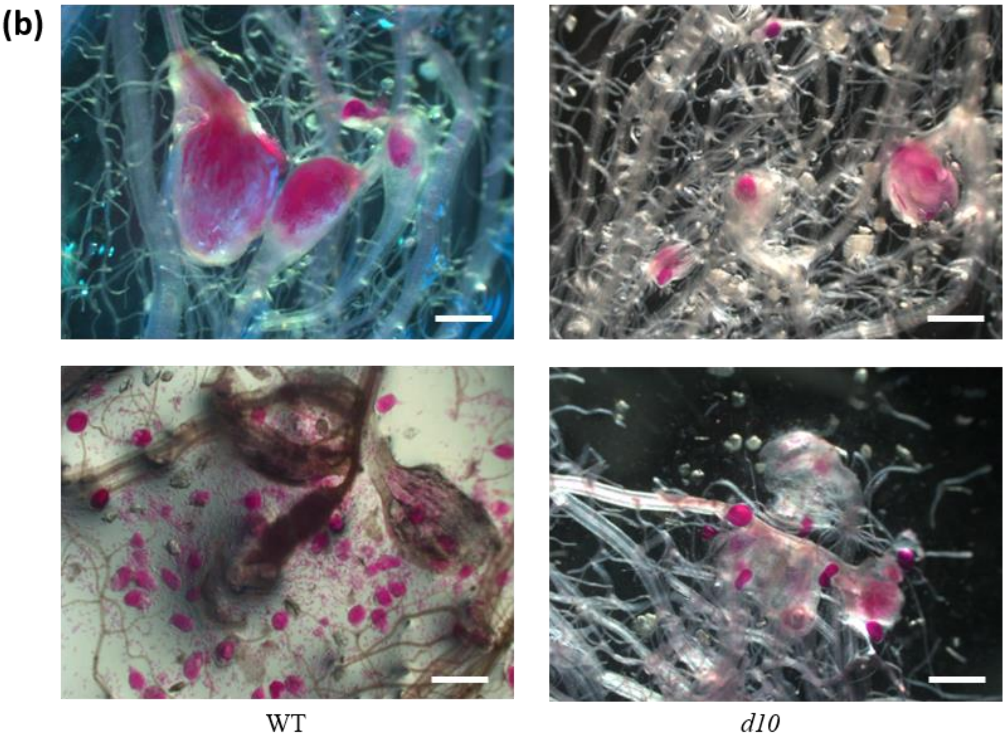

Fig. S4 Percentages of *M. graminicola* induced galls of different sizes in rice *d* mutants. The mutant *d10* and *d27* are SL deficient, while *d14* is an SL signaling mutant. Each two-week-old rice seedling was inoculated with around 200 second stage juveniles (J2) of *M. graminicola*. Samples were collected at 14 dpi, stained in acid fuchsin. The galls were classified in four categories (a) as described by Kyndt *et al*. (2016). Percentage data were analyzed using a Chi-squared test (p = 0.001). (b) Representative pictures of galls of wild-type (WT) and *d10*; upper panel shows intact galls and lower panel shows nematodes after dissecting the galls. Scale bars represent 1 mm.

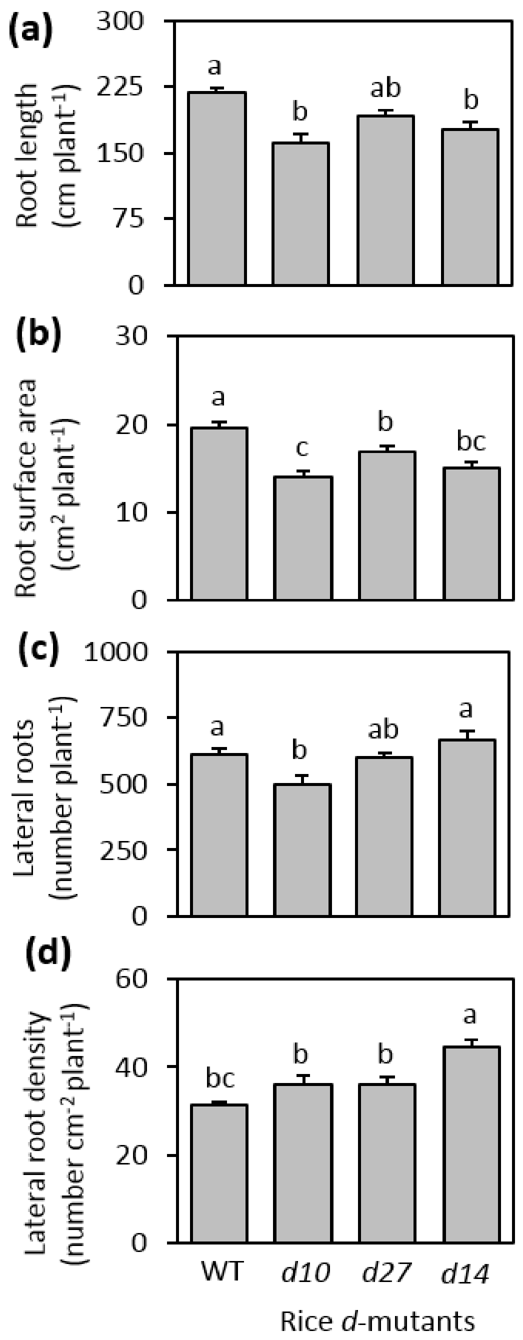

Fig. S5 Root system architectures of rice *d*-mutants and the corresponding wild type plants. The root phenotype of 14-day-old rice seedlings was digitalized using Rhizoscanner and then images were analyzed using the software WinRHIZO to measure total **(a)** root length, **(b)** root surface area, **(c)** number of lateral roots, and **(d)** lateral root density per plant. Data were analyzed by one-way ANOVA followed by Tukey's post-hoc test. Different letters indicate statistically different means at 95% confidence. Data represent mean + SE (n=8).

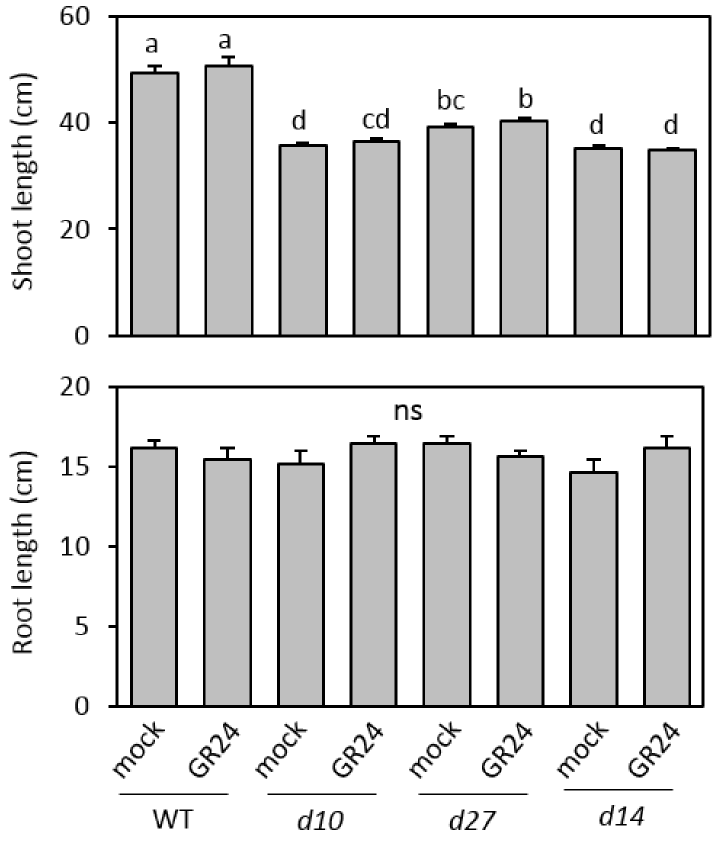

Fig. S6 Effect of foliar GR24 application followed by *M. graminicola* infection on shoot and root lengths of rice *d*-mutants and wild type plants. The mutant *d10* and *d27* are SL deficient while *d14* is an SL signaling mutant. The two-week-old rice seedlings were sprayed with GR24 (0.1 µM) and their respective mock plants were sprayed with solvent only. After 24 hours, each plant was inoculated with around 200 second stage juveniles (J2) of *M. graminicola*. Shoot and root lengths were measured at 14 dpi. Data were analyzed by one-way ANOVA followed by Tukey's post-hoc test. Different letters indicate means were statistically different at 95% confidence. Data represent mean + SE (n=6). ns = not significant.

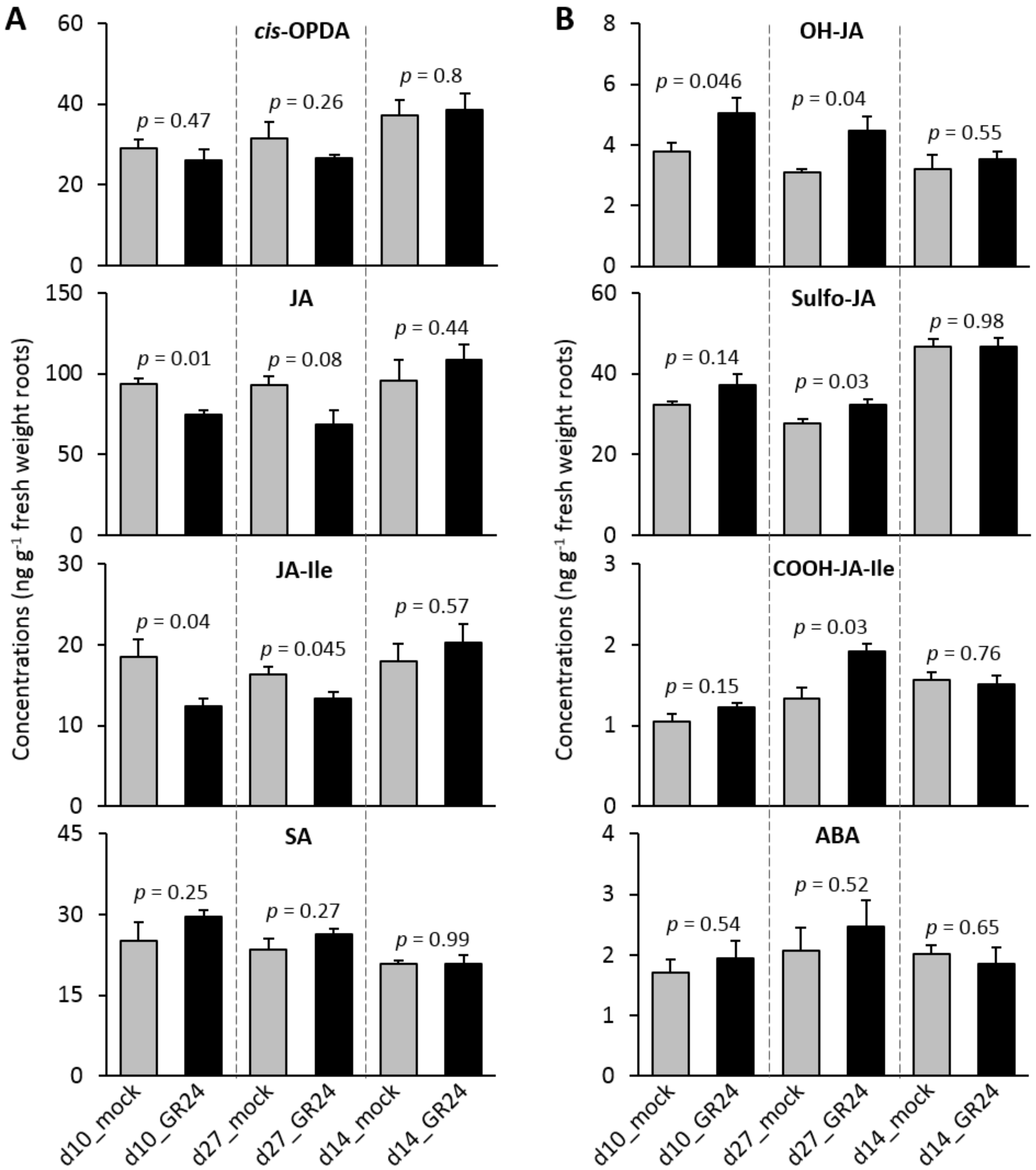

Fig. S7 Hormone metabolites in roots of the rice *d*-mutants after GR24 application. The mutant *d10* and *d27* are strigolactone biosynthetic mutants while *d14* is a signaling mutant. The two-week-old rice seedlings were sprayed with GR24 (0.1 µM) and their respective mock plants were sprayed with solvent only. After 1 day, roots were collected and hormones were analyzed by liquid chromatography - tandem mass spectrometer (LC-MS/MS). Data were analyzed by a two-tailed Student's t-test. Respective *p*-values are indicated on graphs. Data represent mean + standard error (n=4) and each replicate was a pool of 4-6 plants. *cis*-OPDA = *cis*-(+)-12-oxo-phytodienoic acid, JA = jasmonic acid, JA-Ile **=** JA-isoleucine, SA = salicylic acid, OH-JA = hydroxy-JA, COOH-JA-Ile = carboxy-JA-Ile, ABA= abscisic acid.

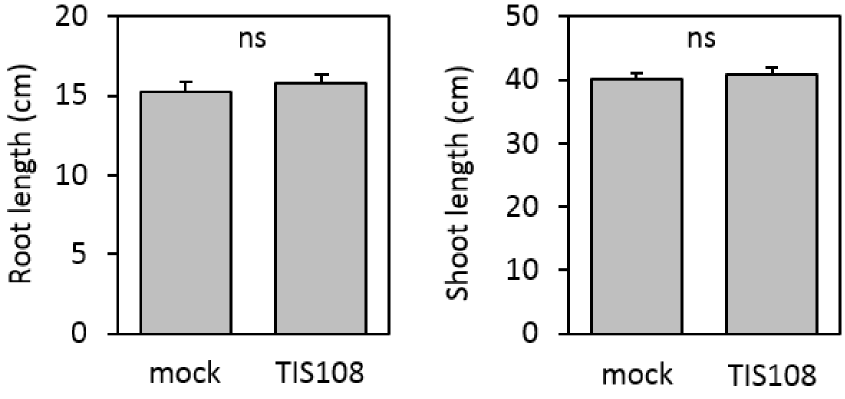

Fig. S8 Effect of foliar TIS108 application followed by *M. graminicola* infection on shoot and root lengths of rice plants. Two-week-old wild type rice seedlings were sprayed with strigolactone biosynthesis inhibitor TIS108 (3 µM) or with solvent only (mock). After 24 hours, each plant was inoculated with around 200 second stage juveniles of *M. graminicola*. Shoot and root lengths were measured at 14 dpi. Data were analyzed by a two-tailed Student's t-test (ns = not significant). Data represent mean + SE (n=8).

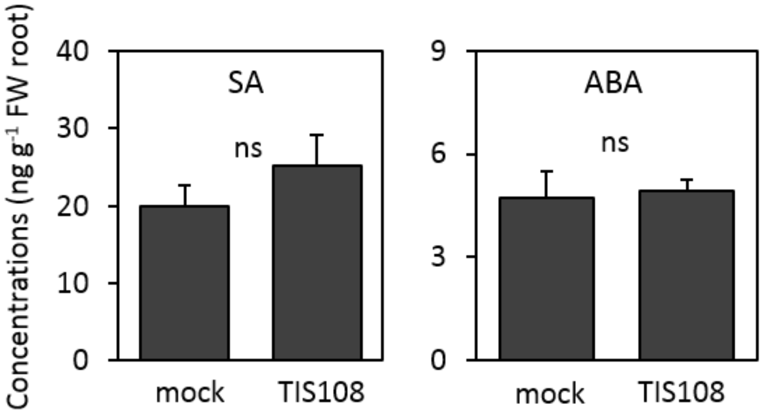

Fig. S9 Effect of foliar application of strigolactone biosynthesis inhibitor (TIS108) on salicylic acid and abscisic acid contents. Two-week-old wild type rice seedlings were sprayed with 0.1 µM TIS108 and their respective mock plants were sprayed with solvent only. After 24 hours, the roots were harvested. SA and ABA content were measured using LC-MS/MS. Data were analyzed by a two-tailed Student's t-test (ns = not significant). Data represent mean + SE (n=5) and each replicate was a pool of 4-6 plants.

Table S1 Primer sequences used in this study for qRT-PCR

Primer	Sequence 5'-3'	Reference
OsD27-for	TCTGGGCTAAAGAATGAAAAGGA	Ito et al., 2017
OsD27-rev	AGAGCTTGGGTCACAATCTCG	Ito et al., 2017
OsD17-for	CGAAGGGAAAGGAGTGGC	Sun et al., 2014
OsD17-rev	CGAACGGGAAGTACGGGAG	Sun et al., 2014
OsD10-for	AGATTGTGGCGAGCGTGGAG	Sun et al., 2014
OsD10-rev	AGGAGCGGAGGTTGTGGAGG	Sun et al., 2014
OsD14-for	TTGAACGACAGCGACTACCACG	Sun et al., 2014
OsD14-rev	GAAGAGGGTGCGGCTGAACT	Sun et al., 2014
OsExp-for	TGTGAGCAGCTTCTCGTTTG	Nahar et al., 2013
OsExp-rev	TGTTGTTGCCTGTGAGATCG	Nahar et al., 2013

References

Ito S, Yamagami D, Umehara M, Hanada A, Yoshida S, Sasaki Y, Yajima S, Kyozuka J, Ueguchi-Tanaka M, Matsuoka M, et al. 2017. Regulation of Strigolactone Biosynthesis by Gibberellin Signaling. *Plant Physiology* **174**(2): 1250-1259.

Kyndt T, Goverse A, Haegeman A, Warmerdam S, Wanjau C, Jahani M, Engler G, de Almeida Engler J, Gheysen G. 2016. Redirection of auxin flow in Arabidopsis thaliana roots after infection by root-knot nematodes. *Journal of Experimental Botany* **67**(15): 4559-4570.

Nahar K, Kyndt T, Hause B, Höfte M, Gheysen G. 2013. Brassinosteroids suppress rice defense against root-knot nematodes through antagonism with the jasmonate pathway. *Molecular Plant-microbe Interactions* **26**(1): 106-115.

Sun H, Tao J, Liu S, Huang S, Chen S, Xie X, Yoneyama K, Zhang Y, Xu G. 2014. Strigolactones are involved in phosphate-and nitrate-deficiency-induced root development and auxin transport in rice. *Journal of Experimental Botany* **65**(22): 6735-6746.
